# Inhibitory neuron migration and IPL formation in the developing zebrafish retina

**DOI:** 10.1242/dev.122473

**Published:** 2015-08-01

**Authors:** Renee W. Chow, Alexandra D. Almeida, Owen Randlett, Caren Norden, William A. Harris

**Affiliations:** 1 Department of Physiology, Development and Neuroscience, University of Cambridge, Cambridge CB2 3DY, UK; 2 MPI of Molecular Cell Biology and Genetics, Pfotenhauerstraße 108, Dresden 01307, Germany

**Keywords:** Amacrine cells, Displaced amacrine cells, Horizontal cells, Inner plexiform layer, Neuronal migration

## Abstract

The mature vertebrate retina is a highly ordered neuronal network of cell bodies and synaptic neuropils arranged in distinct layers. Little, however, is known about the emergence of this spatial arrangement. Here, we investigate how the three main types of retinal inhibitory neuron (RIN) – horizontal cells (HCs), inner nuclear layer amacrine cells (iACs) and displaced amacrine cells (dACs) – reach their specific laminar positions during development. Using *in vivo* time-lapse imaging of zebrafish retinas, we show that RINs undergo distinct phases of migration. The first phase, common to all RINs, is bipolar migration directed towards the apicobasal centre of the retina. All RINs then transition to a less directionally persistent multipolar phase of migration. Finally, HCs, iACs and dACs each undergo cell type-specific migration. In contrast to current hypotheses, we find that most dACs send processes into the forming inner plexiform layer (IPL) before migrating through it and inverting their polarity. By imaging and quantifying the dynamics of HCs, iACs and dACs from birth to final position, this study thus provides evidence for distinct and new migration patterns during retinal lamination and insights into the initiation of IPL formation.

## Abstract (precis)

**Highlighted article:** The quantification of cellular behaviour in real time provides new insights into interneuron migration and inner plexiform layer formation during the lamination of the zebrafish retina.

## INTRODUCTION

Most neurons are born at a distance from the place where they fulfil their function in the mature organism, yet we know little about how neurons migrate to the correct position in one of the simplest laminated neural tissues, the retina. Here, we focus on the movements of RINs – HCs, iACs and dACs – the cell bodies of which are arranged in three distinct layers in the mature retina.

Early studies using electron microscopy and Golgi staining (Gallego, 1986; Génis Galvez et al., 1977; Hinds and Hinds, 1979, 1983; Prada et al., 1987; Tarrés, 1984) demonstrated that presumptive migrating RINs can have both bipolar or multipolar morphologies, and suggested a number of distinct hypotheses relating these morphologies to RIN type and migratory phase. Recently, the development of cell-specific markers has eliminated some of these hypotheses. It was revealed that HCs first undergo basal migration before turning around and migrating apically to their final positions in the inner nuclear layer (INL), that many HCs divide within the INL to give rise to two daughter HCs (Edqvist and Hallböök, 2004; Fard et al., 2013; Godinho et al., 2007; Huckfeldt et al., 2009; Poche et al., 2007; Weber et al., 2014), and that at least some iACs are multipolar before stratifying into the IPL (Deans et al., 2011; Godinho et al., 2005). However, as studies investigating RIN migration have relied mainly on fixed samples or time-lapse movies of short duration, many uncertainties remain regarding the migratory modes of different RIN types (supplementary material Fig. S1).

It is currently unclear how iACs and dACs separate and end up polarizing oppositely on either side of the IPL. Birth-dating studies consistently show that retinal ganglion cells (RGCs), ACs and bipolar cells (BCs) are born in an overlapping sequence, and a study has shown that dACs are born earlier than their equivalently stained iAC counterparts (Voinescu et al., 2009). This has led to the hypothesis that AC lamination results from the stacking of migrating cells, with dACs migrating through an incomplete IPL into the GCL (Rapaport, 2006; Voinescu et al., 2009) (supplementary material Fig. S2A). By staining for immature starburst ACs in the developing chick retina, Spira et al. (1987) formed an alternative hypothesis in which iACs and dACs separate by the emergence of processes directed towards each other, and it is this plexus of AC processes that initiates IPL formation. Recent time-lapse observations are consistent with this idea (supplementary material Fig. S2B;Huberman et al., 2010). However, it has been difficult to test this hypothesis directly by following iACs and dACs during the earliest stages of IPL formation, partly because there are no known markers to distinguish iACs and dACs.

The fact that an IPL can emerge in the complete absence of ACs and various other retinal cell types (Green et al., 2003; Kay et al., 2004; Randlett et al., 2013; Wang et al., 2001) leads to profound questions about how the IPL emerges. Clearly, how the IPL normally initiates and how sublaminae within the IPL begin to form can be only answered by detailed time-lapse studies.

To create a more complete view of RIN migration and IPL formation, we performed extensive time-lapse imaging in the developing zebrafish embryo using a variety of transgenic lines over long periods of time. In this work, the term ‘migration’ refers to all movements of a cell from one position to another, and includes movements due to passive displacements and somal relocations. For each RIN type, we were able to dissect distinct phases of migration with particular attributes. The vast majority of RINs undergo bipolar migration to the middle of the retina before transitioning to multipolar morphologies. By imaging ACs and their emerging processes simultaneously with BCs or RGCs, we have also been able to unravel the sequence of events leading to the separation of iACs and dACs by the IPL, and in doing so, provide new insights into the initiation and sublamination of the IPL.

## RESULTS

### RINs undergo bipolar migration to the apicobasal centre of the retina

**Fig. 1.**
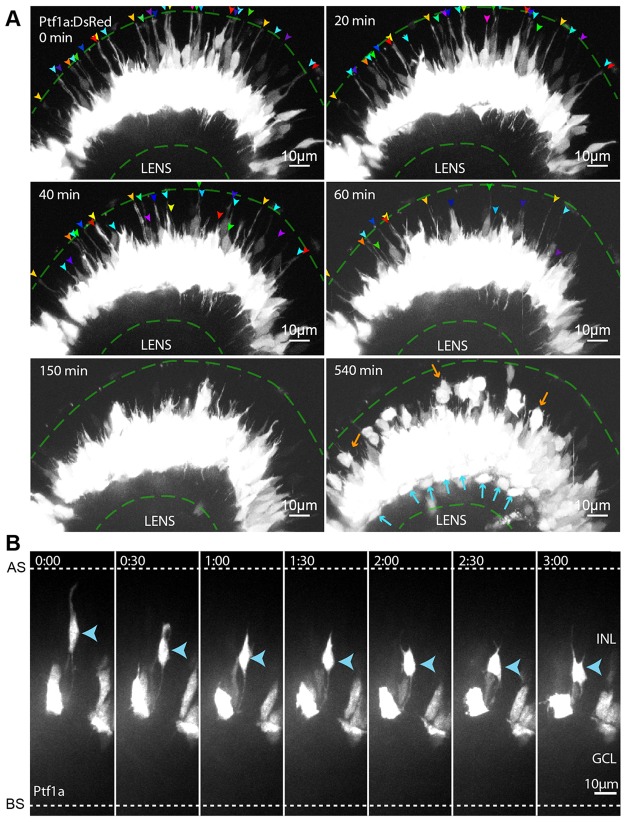
**Basally migrating RINs in apical regions of the retina appear bipolar.** (A) Selected frames from a movie of a Ptf1a:DsRed retina starting at ∼45 hpf. Images are shown as maximum intensity *z*-projections. At 0 min, tips of RIN apical processes (arrowheads) are seen to be attached to the apical surface of the retina (top dashed green line). At 540 min, when dACs (blue arrows) can be seen to separate from the main body of RINs in the middle of the retina, multipolar RINs, presumably HCs, can be seen migrating towards the OPL (orange arrows). (B) Selected time frames from a movie of Ptf1a:DsRed cells transplanted into a WT embryo starting at ∼45 hpf. Blue arrowheads indicate a RIN that transitions from bipolar to multipolar morphology. AS, apical surface; BS, basal surface.

To visualize RIN migration, we made use of a transgenic zebrafish line in which the promoter of the gene that encodes the basic helix-loop-helix (bHLH) transcription factor Ptf1a drives the expression of DsRed in RINs (Jusuf and Harris, 2009). This allowed us to follow several Ptf1a^+^ cells located in apical parts of the retina (Fig. 1A; supplementary material Movie 1). All of these cells exhibited elongated bipolar morphologies as they migrated basally towards the centre of the retina (Fig. 1A, 0 min). Moreover, it became clear that most of these cells retracted their apical processes only after they reached the middle of the retina (Fig. 1A, 150 min). Multipolar Ptf1a^+^ cells in the apical part of the INL (i.e. HCs) became apparent only later (Fig. 1A, 540 min). These observations suggested that many RINs begin their migration in a bipolar mode before switching to a multipolar morphology.

To visualize RIN morphology at the single-cell level, we imaged Ptf1a-labelled cells transplanted into a wild-type (WT) background. We tracked >100 Ptf1a-expressing cells that transitioned from bipolar to multipolar morphology (Fig. 1B). Of these, 44 were tracked long enough to determine their cell type based on morphology and position. Six of these cells were HCs, 26 were iACs and 12 were dACs, indicating that this bipolar-to-multipolar transition occurs in all RINs.

**Fig. 2.**
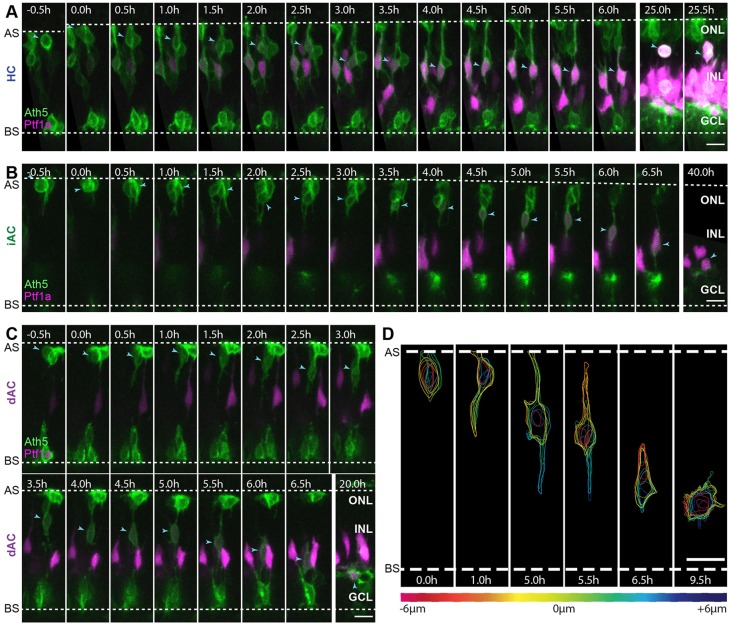
**RINs transition directly into bipolar morphologies after birth.** (A-C) Selected frames of movies starting at ∼44 hpf showing an HC, an iAC and a dAC (indicated by blue arrowheads) from birth. (D) Contour maps of the dAC shown in C at several time points. The dAC was traced *z*-slice by *z*-slice. Each trace was coloured based on the position of the *z*-slice relative to the centre of the cell. *z*-slices are spaced 1 µm apart. Time is shown as hours relative to cell birth. Scale bars: 10 µm. AS, apical surface; BS, basal surface; ONL, outer nuclear layer.

As the Ptf1a:DsRed signal only became visible postmitotically, an alternative strategy was needed to observe the earliest stages in RIN migration. The bHLH transcription factor Ath5 (Atoh7 – Zebrafish Information Network) is expressed during the G2 phase of the final cell cycle in most RINs (Poggi et al., 2005). Therefore, we transplanted cells from Ptf1a:DsRed; Ath5:gapGFP or Ptf1a:GFP;Ath5:gapRFP transgenic lines into WT embryos. We followed 25 Ath5^+^ and Ptf1a^+^ cells from their time of birth. Of these cells, 23/25 were born at the apical surface of the retina (Fig. 2A-C), and two cells were born near the outer plexiform layer (OPL) (supplementary material Fig. S3). All of these cells became elongated in the apical-basal direction immediately after birth (Fig. 2A-C; supplementary material Fig. S3). More than two processes may extend from the cell body, but one or two dominant processes are always aligned in the apical or basal direction such that the cells appear bipolar or unipolar (Fig. 2D; supplementary material Movie 2, 5.0 h). Sixteen of these cells were tracked long enough to identify their cell type based on their eventual morphology, position and, in the case of HCs, a secondary division. Four were HCs (Fig. 2A; supplementary material Movie 3), eight were iACs (Fig. 2B; supplementary material Movie 4) and four were DACs (Fig. 2C; supplementary material Movie 5). These experiments confirmed that early phase migration in all RINs is associated with bipolar morphology.

### HCs, iACs and dACs separate into three distinct locations by multipolar migration

**Fig. 3.**
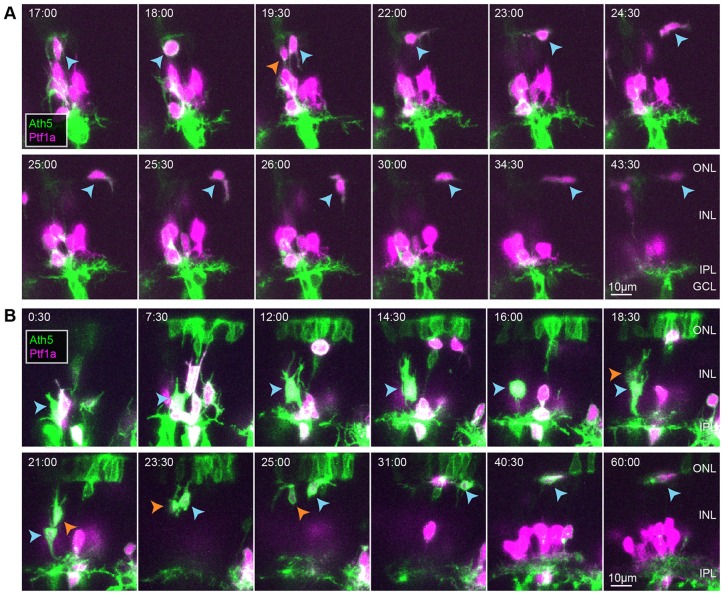
**HCs transition to flattened morphologies and migrate tangentially at regions near the future OPL.** (A,B) Two examples of HCs dividing after migrating basally to the middle of the retina. Time shown in h:min relative to the start of the movie, ∼50 hpf. Blue arrowheads indicate the HC being tracked. Orange arrowheads indicate its sister after terminal division.

During the next phase of migration all RIN types become multipolar. We found that multipolar HCs intermingle with ACs in the basal-most regions of the INL before migrating apically where they divide either adjacent to the forming OPL (10/16 cells; Fig. 3A; supplementary material Movie 3), or en route to the forming OPL (6/16 cells; Fig. 3B). At the region near the future OPL, HCs flatten and often migrate tangentially (*n*=22 cells; Fig. 3). Although HCs in the mature retina are always found located at the apical side of the INL with tangential processes extending from the apical side of the cell, immature HCs sometimes migrate into the photoreceptor layer and have tangential processes extending from the basal side of the cell (5/22 cells; Fig. 3A, 25.0 h, 30.0 h). These observations corroborate many of the findings from previous studies in mice using fixed tissue (Huckfeldt et al., 2009) and studies in zebrafish using high temporal resolution movies spanning shorter time periods (Godinho et al., 2007; Weber et al., 2014).

**Fig. 4.**
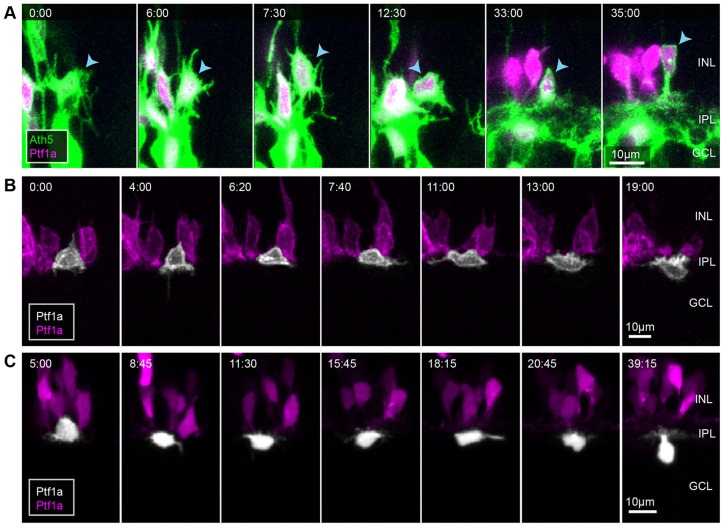
**iACs and dACs undergo a stereotypical sequence of behaviours to polarize and migrate to their respective cell layers.** (A) Example of an iAC (blue arrowhead) stratifying in the INL. (B,C) Two examples of dACs (white) migrating into the GCL. Times are shown in h:min relative to the start of the movies, ∼48 hpf.

We next turned our attention to iACs. We followed 50 iACs throughout the multipolar phase (Fig. 4A; supplementary material Movie 4). Consistent with a previous zebrafish study (Godinho et al., 2005), multipolar iACs appear to stabilize processes that are in contact with the forming IPL (*n*=50 cells; Fig. 4A; supplementary material Movies 4 and 5). During stratification, some of these cells were seen to extend short, tangentially directed protrusions and shuffle their somas tangentially (supplementary material Fig. S4A). Out of the 29 iACs we followed with somas situated one or more cell body diameters apical of the IPL at the end of the imaging session, all were seen at least once during the time-lapse period to be directly adjacent to the IPL, only to move away from the IPL later in the movie (Fig. 4A). Thus, it is not the case that iACs simply pile up over the IPL according to their time of arrival as suggested previously (Rapaport, 2006; Voinescu et al., 2009).

Finally, we examined the behaviour of dACs. We tracked 36 dACs as they migrated into the GCL. The majority (33/36 cells) of these cells were seen to undergo a stereotypical sequence of morphological changes (Fig. 4B,C; supplementary material Movies 6 and 7). We found that multipolar migration brings these cells near the IPL, where they begin to flatten the basal edge of their soma. As the cells flatten and move basally, rapid extension and retraction of their processes becomes predominantly localized along a plane basal to that of simultaneously stabilizing iAC processes. Some dACs were seen to migrate tangentially at this stage, in a manner reminiscent of tangential migration of HCs at the OPL (supplementary material Fig. S4B). dACs then shift their somas further basally and eventually establish an apically directed, bushy arbour. As the cell body rounds up, the bushy arbour matures into a dendritic tree (Fig. 4C; supplementary material Movies 6 and 7). Of these dACs, 18/32 could be followed long enough to see them develop a short stalk (Fig. 4C; supplementary material Movie 7). A minority (3/36 cells) of dACs that we saw did not follow this sequence. These dACs enter the last phase of migration much later than other dACs, and, rather than flattening their cell bodies, they send out a basal process through the proto-IPL to the plane where other dACs have stratified earlier and then rapidly squeeze through the proto-IPL (supplementary material Fig. S5). These findings suggest that dACs actively migrate into the GCL, instead of simply being trapped there by an IPL that forms apically to their somas as previously suggested (Génis Galvez et al., 1977; Rapaport, 2006; Rapaport et al., 2004).

### Radial dynamics of RINs during different phases of migration

**Fig. 5.**
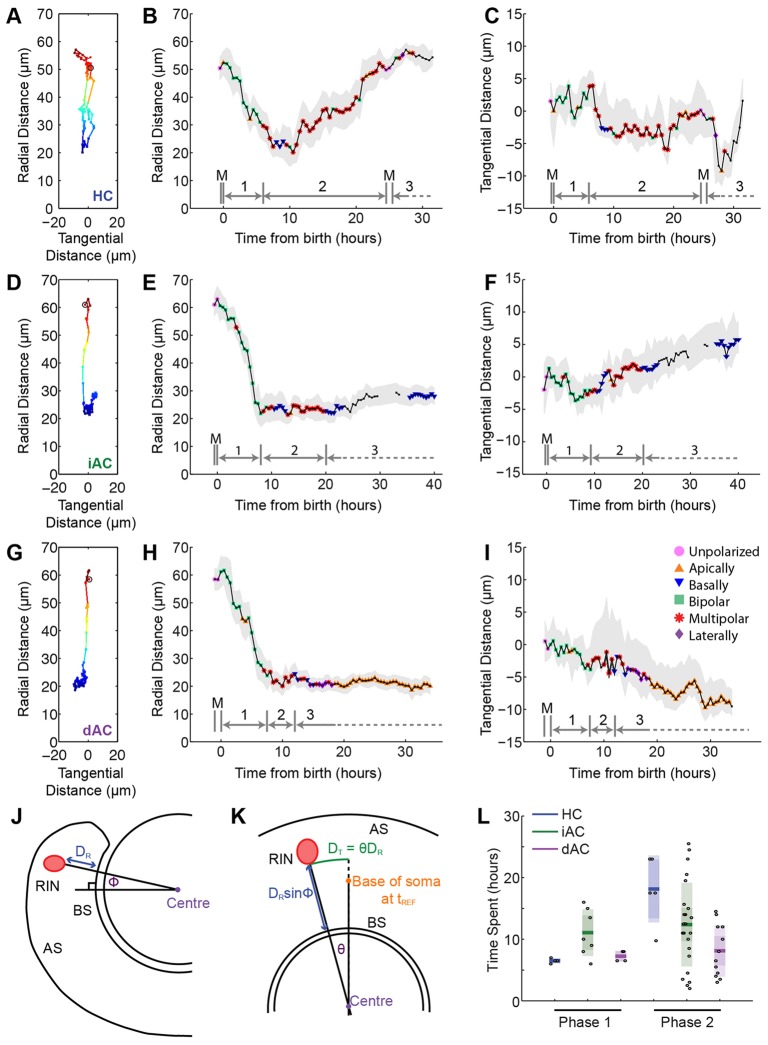
**RIN migration can be separated into distinct phases.** (A-I) A-C show the tracking data for the HC shown in Fig. 2A; D-F show the tracking data for the iAC shown in Fig. 2B; and G-I show the tracking data for the dAC shown in Fig. 2C. (A,D,G) Radial position of the base of the cell relative to the basal surface of the retina and tangential position of the base of the cell relative to its position at birth. The black open circle indicates the start of the track. (B,E,H) Radial position of the cell over time. The greyed area indicates the distance of the apical and basal extent of the cell soma. The black line indicates the distance of the middle of the cell soma from the basal surface. (C,F,I) Tangential position of the cell over time. The greyed area indicates the distance of the lateral extent of the cell soma. The black line indicates the position of the base of the cell soma. For B,C,E,F,H,I, different markers on the black line indicate that the cell morphology was classified at that time point. Above the *x*-axis, grey bars show how different parts of the track were split into different phases: mitosis (M), Phase 1 (1), Phase 2 (2) and Phase 3 (3). (J,K) Schematics of dorsal (J) and lateral (K) views of the retina, showing how a spherical coordinate system was set up and radial (D_R_) and tangential (D_T_) distances were measured (see Materials and methods). AS, apical surface; BS, basal surface. (L) Boxplot showing the mean, 95% confidence interval and one standard deviation of the time RINs spend in Phase 1 and Phase 2.

We next analysed the dynamics of migration. To do this, we tracked RINs in movies taken at a temporal resolution of 15-30 min/time point and split the migration of RINs into three separate phases (Fig. 5; supplementary material Movies 3-5): Phase 1, the unipolar or bipolar period of RIN migration; Phase 2, the period of migration during which RINs are mostly multipolar; and Phase 3, the period of migration following Phase 2, which has no clear morphological commonality among the three types of RIN. For HCs, Phase 3 migration begins when the cells undergo terminal division, for iACs, Phase 3 migration begins at the point when the cells have pruned processes until they have only a single primary dendrite, and for dACs, Phase 3 migration begins when the cells flatten the basal side of their soma in the region of the forming IPL. For the analysis of Phase 1 and Phase 2 cells, only tracks covering the entire phase were used. As there is no obvious endpoint to Phase 3, we confined our analysis to its first 12 h.

**Fig. 6.**
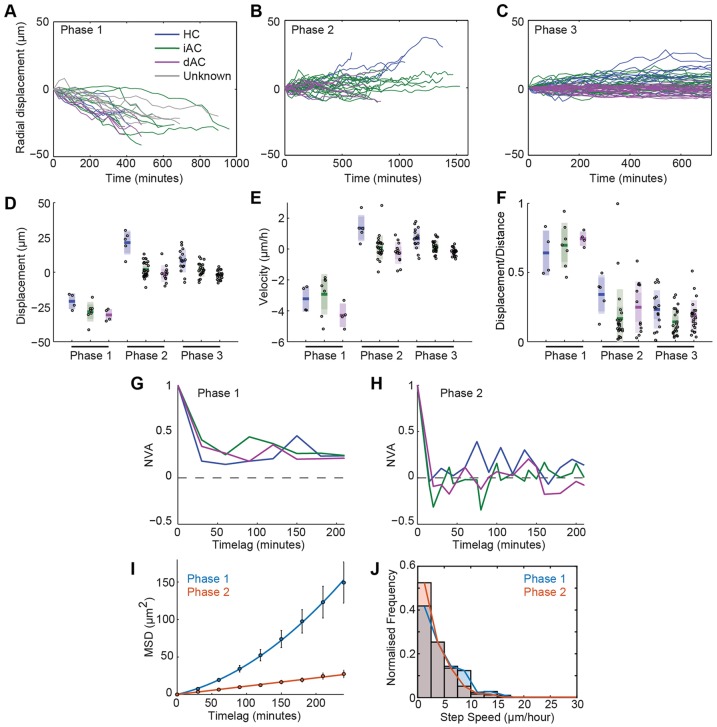
**Radial migration.** (A-C) Radial component of cell tracks of HCs (blue), iACs (green), dACs (magenta) and unclassified inhibitory neurons (grey) for each migration phase. (D-F) Boxplots showing the mean, 95% confidence interval and one standard deviation for displacement (D) and velocity (E) and the ratio of displacement over total distance travelled (F). (G,H) Normalized velocity autocorrelation (NVA) against time lag for Phase 1 (G) and Phase 2 (H). (I) One-dimensional MSD curves for radial migration with a diffusion-flow model fitted to Phase 1 (blue line) and a diffusion model fitted to Phase 2 (orange line). Error bars represent s.e. (J) Histogram of step-speed for Phase 1 and Phase 2.

We first investigated the length of time different RINs spent in Phase 1 and Phase 2. HCs, iACs and dACs spend an average of 6.5 h (σ=0.4 h, *n*=4), 10.7 h (σ=3.6 h, *n*=7 cells) and 7.3 h (σ=0.9 h, *n*=4 cells), respectively, in Phase 1. For Phase 2, HCs, iACs and dACs spend 18.2 h (σ=5.4 h, *n*=5 cells), 12.5 h (σ=6.7 h, *n*=23 cells) and 8.1 h (σ=4.2 h, *n*=11 cells), respectively (Fig. 5). We next analysed the radial component of RIN migration (Fig. 6). RINs migrating in Phase 1 cover an average distance of 24.6 µm (σ=7.5 µm, *n*=22 cells) basally (Fig. 6D). The differences in the distance covered by the various RIN types in Phase 1 are not statistically significant (rank-sum test). However, during Phase 2, HCs migrate 21.3 µm (σ=8.7 µm) apically, whereas iACs and dACs migrate only 1.8 µm (σ=5.9 µm) apically and 1.1 µm (σ=6.1 µm) basally, respectively (Fig. 6D). During the first 12 h of Phase 3, HCs, iACs and dACs migrate 8.2 µm (σ=8.3 µm) apically, 1.7 µm (σ=4.9 µm) and 2.1 µm (σ=3.0 µm) basally, respectively. Thus, it appears that Phase 1 migration is primarily responsible for delivering RINs into the centre of the retina, which for ACs is close to their final laminar position (Fig. 6D), but not for HCs, which migrate apically through the developing INL during Phase 2 and the early part of Phase 3.

The velocities of radial migration in Phase 1 for HCs, iACs and dACs are 3.1 µm/h (σ=0.8 µm/h), 2.9 µm/h (σ=1.3 µm/h) and 4.3 µm/h (σ=0.7 µm/h), respectively. During Phase 2, HCs, iACs and dACs have much slower average speeds [1.4 µm/h (σ=0.9 µm/h), 0.5 µm/h (σ=0.6 µm/h) and 0.6 µm/h (σ=0.5 µm/h), respectively], and this slows down even further during the first 12 h of Phase 3, in which HCs, iACs and dACs have average speeds of 0.8 µm/h (σ=0.5 µm/h), 0.3 µm/h (σ=0.3 µm/h) and 0.3 µm/h (σ=0.2 µm/h), respectively (Fig. 6E). As the greater average speeds of RINs in Phase 1 compared with Phase 2 could be due to differences in directional persistence or in instantaneous speed, we first compared directional persistence in the radial direction of cells in Phase 1 and Phase 2 using a simple measure of the ratio of displacement over total distance travelled, and found that the ratio was significantly higher in Phase 1 than in Phase 2 (rank-sum test; Fig. 6F). We then used a one-dimensional velocity autocorrelation analysis, and found that the normalized velocity autocorrelation function always lies above zero for all RIN types in Phase 1, but takes both positive and negative values for iACs and dACs, and mostly positive values for HCs during Phase 2, suggesting that only HCs display directional persistence (apical) in Phase 2 (Fig. 6G,H). Greater directional persistence is also evident from the one-dimensional mean square displacement (MSD) curves, in which averaged Phase 1 MSD values plotted against time lag appear to display a curvilinear trend, whereas averaged Phase 2 MSD values plotted against time lag appear to display a linear trend (Fig. 6I). We tested the MSD curves from the two phases of migration against a diffusion model, a flow model and a diffusion-flow model. Using a Bayesian inference approach (Monnier et al., 2012), we found that tracks of RINs in Phase 1 showed >98% probability for the diffusion-flow model, whereas tracks of RINs in Phase 2, either pooled together or analysed separately by cell type, showed the greatest probability for the diffusion model (pooled: >99%; HCs: 87%; iACs: >99%; dACs: >99%). Obviously, however, HCs must exhibit at least a small amount of apical flow in Phase 2 because they migrate in this direction.

We next investigated whether the greater average velocities of RINs in Phase 1 compared with Phase 2 are solely due to greater directional persistence in Phase 1. To do this, we measured the step-speed of RINs, calculated as the average speed between two consecutive time frames (Fig. 6J). We found that RINs in Phase 1 RINs had an average step speed of 4.1 µm/h, whereas RINs in Phase 2 had a statistically significant lower average step-speed of 3.3 µm/h (*P*<0.0001, rank-sum test). This suggests that cells may have faster instantaneous velocities when they are bipolar than when they are multipolar. However, owing to the fact that we use 15-30 min time intervals, it is also possible that more frequent changes in direction between these points could account for the differences observed.

### Dynamics of tangential migration

**Fig. 7.**
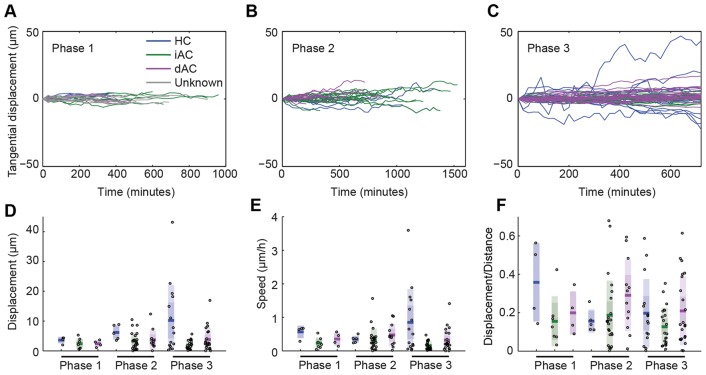
**Tangential migration.** (A-C) Tangential component of cell tracks of HCs (blue), iACs (green), dACs (magenta) and unclassified inhibitory neurons (grey) for each migration phase. (D-F) Boxplots showing the mean, 95% confidence interval and one standard deviation for magnitude of displacement (D), velocity (E) and the ratio of displacement over total distance travelled (F).

Having analysed apico-basal migration, we next investigated the dynamics of tangential migration in the three types of RIN (Fig. 7), as this is crucial for our understanding of how neurons become properly spaced within their respective layers. To begin to answer this question, we analysed the average values for distance and speed for each phase and found that they are not significantly different from one another. However, whereas only 0.05% of RINs migrated >5 µm tangentially, which is roughly one somal length, during Phase 1, 27% of RINs migrated >5 µm in Phase 2 and 22% of RINs migrated >5 µm in the first 12 h of Phase 3, suggesting that tangential migration occurs mainly during later stages of migration. At all phases of migration, cells were seen to migrate tangentially in one direction, only to reverse direction at a later time. This lack of directional persistence often results in low ratios of displacement over distance travelled (Fig. 7F). Thus, it seems that the first bipolar phase of migration is used primarily for getting RINs to the apico-basal centre of the retina, but that the second and third phases of migration are used to refine the position of the cells into specific layers and establish mosaic arrays within these layers.

### IPL formation

**Fig. 8.**
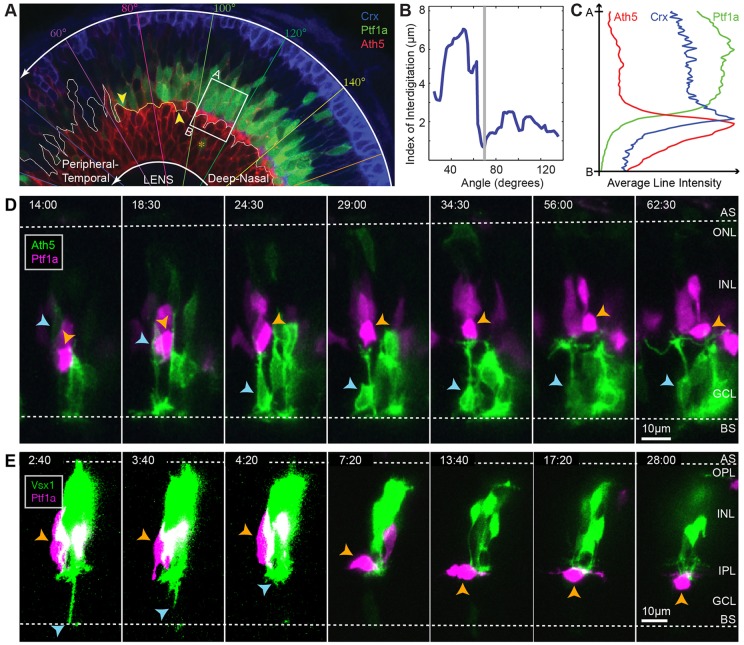
**Early stages of IPL formation.** (A) Snapshot of a 55 hpf retina showing different stages of retinal development along the developmental wave. The RIN labelled with the orange asterisk probably represents a misplaced cell. (B) Quantification of the degree of interdigitation along the apical surface of RGCs, drawn as a white line in A. The vertical grey line indicates the angular position of the minimum value, and the corresponding 20° segment is indicated by the yellow line and yellow arrowheads in A. (C) The average line intensity values of the boxed region in A, showing the BC plexus (Crx) located between the RGC (Ath5) dendritic plexus and RINs (Ptf1a) in this region. (D) Selected frames of a movie of an RGC (blue arrowheads) migrating basally past RINs (orange arrowheads) and stratifying at a location basal to the RINs. (E) Selected frames of a movie of a BC axon (blue arrowheads) retracting to the basal side of RINs in the INL before the subsequent migration of a dAC (orange arrowheads) into the GCL. Time is shown as h:min from the start of the imaging sessions, at ∼44 hpf (D) and ∼48 hpf (E).

Our finding that multipolar dACs migrate basally through a pre-formed proto-IPL (Fig. 4C; supplementary material Fig. S3) does not support current hypotheses on IPL formation (supplementary material Fig. S2). To gain further insights into how the IPL originates, we examined the formation of the IPL across the wave of retinal differentiation in SoFa1 (Almeida et al., 2014) embryos (Fig. 8A). We observed that RINs can first be visualized in the centre of the retina at times when RGCs are still migrating and that RGCs and RINs are initially interdigitated in the GCL. However, consistent with our previous work (Almeida et al., 2014), we find that this interdigitation is lost as development proceeds and the unevenness of the apical side of the RGC layer decreases to a minimum before the first signs of a continuous RGC dendritic plexus (Fig. 8A,B). Shortly after a sharp boundary is formed between RGCs and RINs, a BC (Crx-positive) axonal plexus forms at the interface between RGCs and RINs (Fig. 8A,C), and it is only after the BC plexus begins to form that dACs migrate to their positions within the GCL (Fig. 8A,C). Our analysis also shows that late-arriving RGCs can migrate basally past ACs to take up residence in the GCL (*n*=4 RGCs; Fig. 8D), arguing against the idea that ACs arrive at a pre-formed GCL (Godinho et al., 2005).

Previous studies have shown that BC axons develop from basal processes that retract to the region of the proto-IPL of the retina where they branch (Morgan et al., 2006; Randlett et al., 2013; Weber et al., 2014). To examine the relationship between the BC axonal plexus and the processes of iACs and dACs in more detail, we performed time-lapse imaging of Ptf1a- and Vsx1-labelled cells transplanted into a WT background. Vsx1-driven GFP is initially expressed by a large population of neuroepithelial cells, but later becomes restricted to BC progenitors (Vitorino et al., 2009). Consistent with our static observations in the SoFa1 fish, we see that BC axons initially retract from the basal surface of the retina and elaborate processes at positions basal to RINs (*n*=2 embryos; Fig. 8E, time=4:20) before the migration of dACs through the BC axonal plexus (Fig. 8E, time=13:40 to 28:00). Thus, the IPL in the zebrafish retina begins to form at the interface of RGCs and RINs before the basal migration of dACs through this proto-IPL.

To investigate how iAC and dAC sublaminae within the IPL arise, we first performed static imaging of SoFa2 retinas (Almeida et al., 2014), in which RINs are mosaically labelled. This suggested that during the early stages of IPL formation, when the axonal plexus of BCs lies apical to the RGC dendritic plexus, dAC processes target the interface between the BC axonal plexus and the RGC dendritic plexus, whereas most presumptive iAC processes appeared to target the apical side of the BC plexus (supplementary material Fig. S6). As the IPL matures and RGCs extend processes further into the IPL, iAC and dAC somas appear to move further apart owing to the growing IPL, such that iACs and dACs with somas originally directly adjacent to the BC plexus develop a short stalk (supplementary material Fig. S6). Given that starburst AC circuits are among the first retinal circuits to form (Ford and Feller, 2011), we wondered if the first two AC strata on either side of the BC plexus corresponded to the future S2 and S4 sublamina in the mature retina. We stained these retinas for starburst ACs using an antibody against transcription factor Sox2 (Taranova et al., 2006), and indeed found that INL Sox2^+^ ACs and displaced Sox2^+^ ACs stratified on either side of the BC axonal plexus at this stage (supplementary material Fig. S6D).

**Fig. 9.**
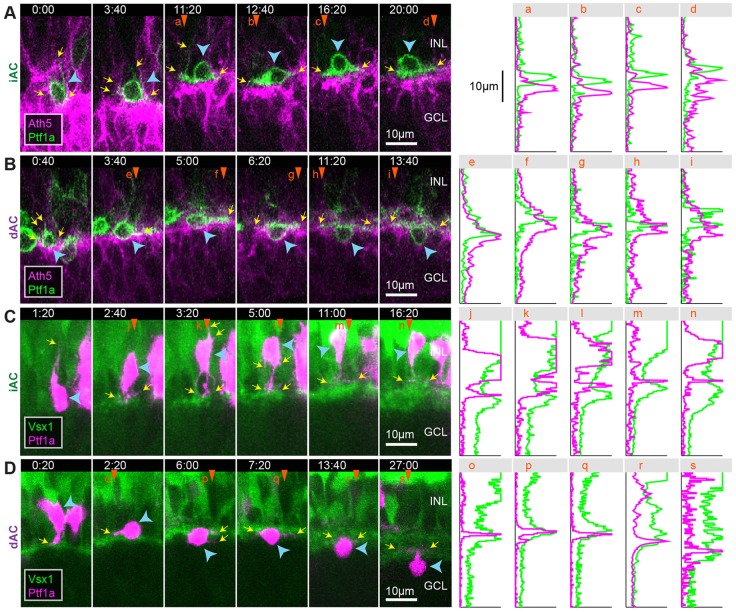
**iACs and dACs differentially stratify within the proto-IPL.** (A,B) Selected frames of a movie of an iAC (A) and a dAC (B) transitioning from multipolar morphology to unipolar morphology in the Ath5 background. (C,D) Selected frames of a movie of an iAC (C) and a dAC (D) transitioning from multipolar morphology to unipolar morphology in the Vsx1 background. Panels on the right indicate the intensity line profile of the corresponding vertical line marked by orange arrowheads. Yellow arrows indicate the tips of the cells' processes. Blue arrowheads indicate cell somas. Time is shown as h:min from the start of the imaging sessions, at ∼50 hpf.

To see how this sublamination occurs in real time, we imaged Ath5:gapRFP;Ptf1a:Gal4;UAS:YFP retinas, in which YFP is mosaically expressed in RINs, to follow the processes iACs and dACs in relation to the RGC plexus. All iACs (5/5) and dACs (6/6) imaged in the background of Ath5:gapRFP preferentially stabilized their processes at a plane apical to the RGC dendritic plexus (Fig. 9A; supplementary material Movie 8). The dACs then migrated through the RGC dendritic plexus (Fig. 9B; supplementary material Movie 9). We then imaged Ptf1a:DsRed;Vsx1:GFP cells that have been transplanted into Vsx1:GFP embryos to see the full BC plexus. Here, 5/6 iACs imaged preferentially stabilized their processes at the apical side of the BC axonal plexus (Fig. 9C; supplementary material Movie 10). The last iAC imaged in the background of Vsx1:GFP appeared to initially target processes to the apical side of the BC plexus, but then preferentially stabilized processes in the middle of the BC plexus, and later matured as a monostratified AC with an arbour that lies between ON and OFF BCs (supplementary material Fig. S7). Although dACs may initially target the apical side of the BC plexus (Fig. 9D, time=0:20-2:20), all dACs imaged (7/7) localized their processes to the basal side of the BC axonal plexus (Fig. 9D, time=7:20; supplementary material Movie 11). The BC axonal plexus eventually expands basally past dAC processes, concomitant with dACs developing a stalk (Fig. 9D; supplementary material Movie 11). We also observed iACs (*n*=2) and dACs (*n*=2) migrating tangentially away from their clonal column after dACs in the area have been separated from iACs by the BC axonal plexus. The processes extending from these cells appear to arborize at, or run tangentially along, the apical or basal side of the BC plexus, respectively (supplementary material Movie 12), suggesting that the proto-IPL might serve as a substrate for tangential migration.

## DISCUSSION

**Fig. 10.**
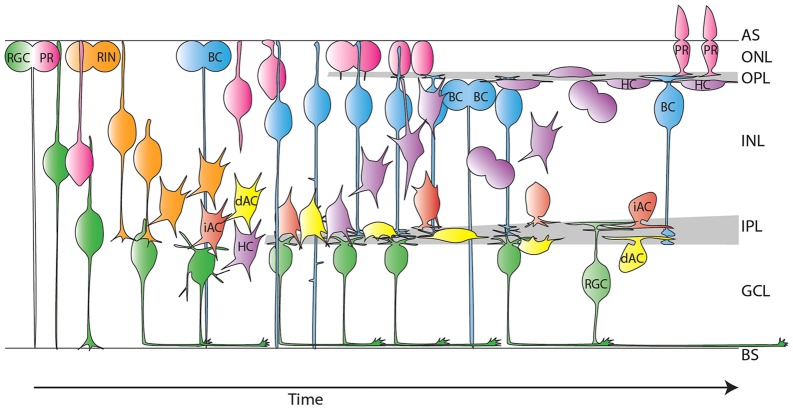
**Model of RIN migration.** All RIN types migrate via bipolar morphologies away from the apical surface before transitioning to multipolar morphology. They gather as a single population in the middle of the retina until the IPL starts to form. HCs then migrate apically and divide en route to, or at, the OPL. HCs sometimes migrate into the outer nuclear layer (ONL) before taking on mature morphology in the INL. iACs stabilize processes at the apical side of the BC plexus, whereas dACs preferentially localize processes to the interface between BC axons and RGC dendrites before moving into the GCL. AS, apical surface; BS, basal surface; PR, photoreceptor. The model shown takes into account results from this study and previous studies (Choi et al., 2010; He et al., 2012; Mumm et al., 2006; Randlett et al., 2013; Suzuki et al., 2013; Weber et al., 2014).

Several new features of RIN migration in the zebrafish have been captured in our time-lapse movies (Fig. 10). All RIN types undergo a first phase of migration during which cells show bipolar cellular morphology and directional persistence. This phase delivers the cells from the apical surface to the centre of the retina. RINs then enter a second phase of migration, characterized by multipolar morphology, tangential migration, and frequent changes of migratory direction. Although both phases last around 10 h, more apicobasal distance is covered during the first phase and more tangential distance is covered in the second phase. The third phase of migration, during which RINs reach their final position, involves fine-tuning of apical-basal position, and, for some cells, a phase during which they travel large distances tangentially. These findings stand in contrast to the view that: (1) HCs migrate by bipolar migration whereas ACs migrate via multipolar migration (Edqvist and Hallböök, 2004) (supplementary material Fig. S1A,C,F); (2) some subtypes of ACs undergo bipolar migration, whereas some subtypes of ACs undergo multipolar migration (Hinds and Hinds, 1983; Link, 2006; Prada et al., 1987) (supplementary material Fig. S1C,D); (3) iACs switch from multipolar to bipolar migration (Deans et al., 2011) (supplementary material Fig. S1E); or (4) all RINs migrate solely by multipolar migration (Randlett et al., 2011a) (supplementary material Fig. S1A,C,F). Instead, our findings seem to be in line with the often forgotten suggestions of Gallego (1986).

The observation that all RINs switch between different modes of migration is consistent with the observations of Noctor et al. (2004), who showed that most cortical neurons born in the ventricular zone switch from bipolar to multipolar modes of migration on their way towards the marginal zone. Our dynamic analysis showing that the speed of migration is faster in the bipolar than the multipolar phase is also consistent with studies in the developing cortex, where it was reported that neurons travel at 10-50 µm/h during bipolar migration but only ∼2 µm/h during multipolar migration (LoTurco and Bai, 2006). However, whereas multipolar neurons are believed to switch to bipolar migration along radial glia in the neocortex, Müller glia are believed to be born too late during retinal development to guide basally migrating RINs, and RIN lamination in retina lacking Müller glia appears unaffected (Randlett et al., 2013). Indeed, our morphological data suggest that RINs initially undergo a form of somal translocation similar to that of young RGCs and photoreceptors (Randlett et al., 2011b; Suzuki et al., 2013; Zolessi et al., 2006). Although RINs have been described to migrate in a strikingly different manner to RGCs, it should perhaps be noted that after axon initiation, RGC dendrites extend from multiple poles of their soma before becoming biased apically (Choi et al., 2010; Kita et al., 2013), and that RGCs in retinas lacking laminin display full multipolar morphology (Randlett et al., 2011b). It will therefore be interesting to explore in future studies the degree to which the two cell types share migratory and polarization mechanisms at a molecular level.

It has long been suspected that tangential migration driven by homotypic repulsion plays a role in mosaic formation of RINs within retinal layers. Our measurements suggesting that tangential migration occurs predominantly at later stages of RIN migration are consistent with previous studies using clonal boundary analysis (Reese and Galli-Resta, 2002; Reese et al., 1995, 1999). It was previously shown that HCs near the proto-OPL can translocate their somas through short tangential processes in a manner suggestive of active tangential migration (Huckfeldt et al., 2009); we now extend these findings to iACs and dACs at the proto-IPL. The fact that we observe iACs and dACs migrating tangentially even after they have been separated into two distinct populations by the BC plexus is consistent with a recent study showing that multiple-EGF-like-domains 10/11 proteins facilitate mosaic formation of both INL and displaced starburst ACs despite the fact that the two mosaics are independent of each other (Kay et al., 2012).

Another novel finding presented here concerns the migration of dACs into the GCL during the third phase of migration. dACs in the INL direct neurites basally, and it is only as they move their somas to positions basal to iACs that their polarities flip. To our knowledge, this is the first study describing a neuron type that inverts its neuritic polarity within a plexus. Our results are in contrast to current hypotheses on IPL formation (supplementary material Fig. S2) and support earlier suggestions that dACs migrate from the INL to the GCL during a specific period of development (Galli-Resta et al., 1997; Schmitt and Dowling, 1999).

The fact that dACs send their process to laminar locations in the proto-IPL that differ from iACs before they become interdigitated with RGCs in the GCL suggests that these cells commit to a dAC fate before they actually become displaced. It is interesting to note that there may be mechanistic links between correct sublaminar stratification of processes in the IPL and the migration of ACs to their correct layer. For example, in Sema6a knockout mice, additional calbindin-positive ACs are found in the GCL (Matsuoka et al., 2011); in Fat3 cadherin knockout mice, Bhlhb5 (Bhlhe22 – Mouse Genome Informatics)-positive ACs are mislocalized to the IPL or the GCL (Deans et al., 2011); and in chicken retinas depleted of DsCam, cholinergic ACs are mislocalized to the IPL (Yamagata and Sanes, 2008).

Recent work on the IPL has focused on how ingrowing neurites choose one or more of many available sublaminae (Nguyen-Ba-Charvet and Chédotal 2014; Robles and Baier 2012; Sanes and Zipursky, 2010), but the question that our work brings to the fore is how sublaminae originate. We suggest that RGCs and RINs create an interface between them that defines where the IPL first forms, that the BC axonal plexus originates at the boundary between RINs and RGC dendrites, and that the processes of dACs and iACs form third and fourth sublaminae on either side of the BC axonal plexus (Fig. 10). One could imagine that repulsive cues initially separate these processes into layers, but that later in development, attractive or adhesive cues are added allowing cells of different subtypes to begin to co-stratify in different combinations, thereby allowing circuit specific connections to form. This idea is not inconsistent with other suggested possibilities that depend on timing and positional information (Becker et al., 2003; Huberman et al., 2010; Morey et al., 2008; Perez and Halfter, 1993; Petrovic and Hummel, 2008).

In summary, we have used time-lapse imaging to provide the first detailed account of RIN migration patterns in the vertebrate retina. In doing so, we have challenged several existing hypotheses for how these cell types move to their appropriate locations in the retina and have provided insights into how the proto-IPL first forms. The next important step to gain further understanding of RIN migration will be to find the molecular motors and mechanisms that drive these events.

## MATERIALS AND METHODS

### Animals

Zebrafish were maintained and bred at 26.5°C, and embryos were raised at 25°C-32°C and staged as described previously (Kimmel et al., 1995) in hours post fertilization (hpf). Embryos were treated with 0.003% phenylthiourea (Sigma) from 20 to 48 hpf and 0.005% phenylthiourea (Sigma) from 48 hpf until the end of the experiment to prevent pigmentation, and were anaesthetized using 0.04% MS-222 (Sigma) prior to mounting.

### Transgenic lines

Transgenic lines used in this study have been previously described: Tg(Ptf1a:GFP) (Godinho et al., 2005), Tg(Ptf1a:DsRed) (Jusuf et al., 2012), Tg(Ath5:gapGFP) and Tg(Ath5:gapRFP) (Zolessi et al., 2006), Tg(Vsx1:GFP) (Kimura et al., 2006; Vitorino et al., 2009), Tg(Ptf1a:Gal4;UAS:YFP) (Godinho et al., 2005), SoFa1 and SoFa2 (Almeida et al., 2014). Double transgenic lines were created by crossing single transgenic lines to each other. For fish that were homozygous, both reporters were screened by mating single fish with WT fish and examining embryos for fluorescence expression.

### Blastomere transplantations

Blastomere transplantations were performed to follow the migration of individual cells. At ∼4 hpf, embryos were dechorionated by 0.3 mg/ml pronase digestion (Sigma) and placed in 1.2% agarose moulds. Blastomeres from donor embryos were transferred into the animal poles of host embryos using a glass capillary connected to a 2 ml syringe.

### Imaging

Embryos were typically collected at ∼48 hpf to capture early stages of AC migration or ∼55 hpf to capture ACs migrating through the BC plexus. Embryos were screened using a fluorescence upright microscope and embedded in 1% low-melting agarose in glass-bottomed microwell dishes (Matek or custom-made) for imaging. Time-lapse imaging was performed using a spinning-disc microscope (PerkinElmer Spinning Disk UltraVIEW ERS) with a 60× water (1.3 NA) objective or an inverted confocal microscope (Olympus FV1000) with a 60× silicon (1.35 NA) objective. Three-dimensional imaging was performed using live embryos (fixation was found to decrease embryo transparency) using an inverted confocal microscope (Olympus FV1000) and 60× silicon (1.35 NA) objective. All cells analysed were examined *z*-slice by *z*-slice at each time point. Minimum temporal resolution of movies was 40 min/time point.

### Software

The following software was used for data analysis and visualization: Volocity (Improvision), FIJI (National Institutes of Health), Matlab (MathWorks), Ndsafir (Boulanger et al., 2007), Photoshop (Adobe Systems), Illustrator (Adobe Systems) and Excel (Microsoft Office).

### Image pre-processing

All datasets were first screened and, if necessary, cropped in Volocity. Coarse drift correction was performed using the Correct 3D Drift plugin in FIJI (Parslow et al., 2014).

### Cell tracking

Quantification of RIN migration was performed for movies taken at a minimum temporal resolution of 30 min/time point. Translational and rotational drift correction in the *xy* plane was performed using control point registration in Matlab. Cell tracking was performed manually using MTrackJ in FIJI (Meijering et al., 2012), where the point of the cell soma closest to the basal surface of the retina was selected. To calculate radial and tangential movements of cells, a spherical coordinate system was set up based on the curvature of the basal surface immediately basal to tracked clones (Matlab). At time points corresponding to approximately every 330 min, we examined 10-20 brightfield images of the *z*-stack and traced the basal surface of the local region. At each of these time points, a sphere was fitted to the basal surface using least squares. We kept the *xy* coordinates of the centre of this fitted sphere to calculate the centre of the spherical coordinate system but discarded the *z* coordinate, due to the difficulty in locating edges of the basal surface in deeper sections of the retina. Assuming that the apical-basal axis of the retina is roughly perpendicular to the basal surface, we placed the centre of the spherical coordinate system for each of these time points on the *z* plane at which the apical-basal axis of the retina was parallel to the *xy* plane. The centre of the spherical coordinate system used was calculated by averaging the centre coordinates taken from each.

Radial distances were calculated as the distance between the tracked point and the centre of the sphere, minus the radius, and tangential distance at any specific time point was measured as the angular displacement multiplied by radial distance at that time point. All graphs were made in Matlab. Statistical differences were calculated using the Wilcoxon rank-sum test. MSD values were calculated using all tracks with time intervals of 30 min or less using MSD Analyzer (Tarantino et al., 2014). For tracks measured at time intervals that were not a factor of 30 min, linear interpolation was used to extract MSD values at multiples of 30 min. MSD curves for each phase of migration were tested against diffusion, flow and diffusion-flow models, with probabilities for each of these models calculated using MSD Bayes software (Monnier et al., 2012). Autocorrelation analysis was performed using MSD Analyzer (Tarantino et al., 2014).

The kinetics data reported in this paper relate to confocal microscopy experiments on immobilized, transgenic fish kept at ∼27°C using a temperature-controlled stage.

### Calculating the index of interdigitation

To calculate the index of interdigitation, we first defined a polar coordinate system by tracing the apical surface of the retina and then fitting a circle, with centre c, defining the origin. We then traced the maximal apical/minimum basal radial position, t(θ), of the cell layer of interest as a function of azimuth, and defined the index of interdigitation, I(θ,Δθ), as the standard deviation of t(θ) over each θ±Δθ segment. In our analysis, we choose Δθ=10°, which results in sufficient samples to extract a meaningful standard deviation of t(θ) while being small enough to observe changes across the retinal wave.

### Data visualization

Average line intensity profiles were made in Matlab (Meijering et al., 2012). For figures and movies, contrast enhancement, noise reduction and pseudo-colouring were performed using a combination of Volocity, Photoshop and Matlab. Addition of arrows/lines/labels within images was performed using Illustrator and FIJI.

## Supplementary Material

Supplementary Material
