# Supplementary material for: Inhibitory neuron migration and IPL formation in the developing zebrafish retina
Source: Development. 2015 Aug 1;142(15):2665–77. doi: 10.1242/dev.122473 (PMC4529032; doi:10.1242/dev.122473)
Supplement: Supplementary Material [file supp_142_15_2665__index.html]

Supplementary Material 

# Inhibitory neuron migration and IPL formation in the developing zebrafish retina

## DEV122473 Supplementary Material

- Supplementary Material
