# Supplementary material for: Inhibitory neuron migration and IPL formation in the developing zebrafish retina
Source: Development. 2015 Aug 1;142(15):2665–77. doi: 10.1242/dev.122473 (PMC4529032; doi:10.1242/dev.122473)
Supplement: Supplementary Material [file supp_dev.122473_DEV122473supp.pdf]

Supplementary Figures

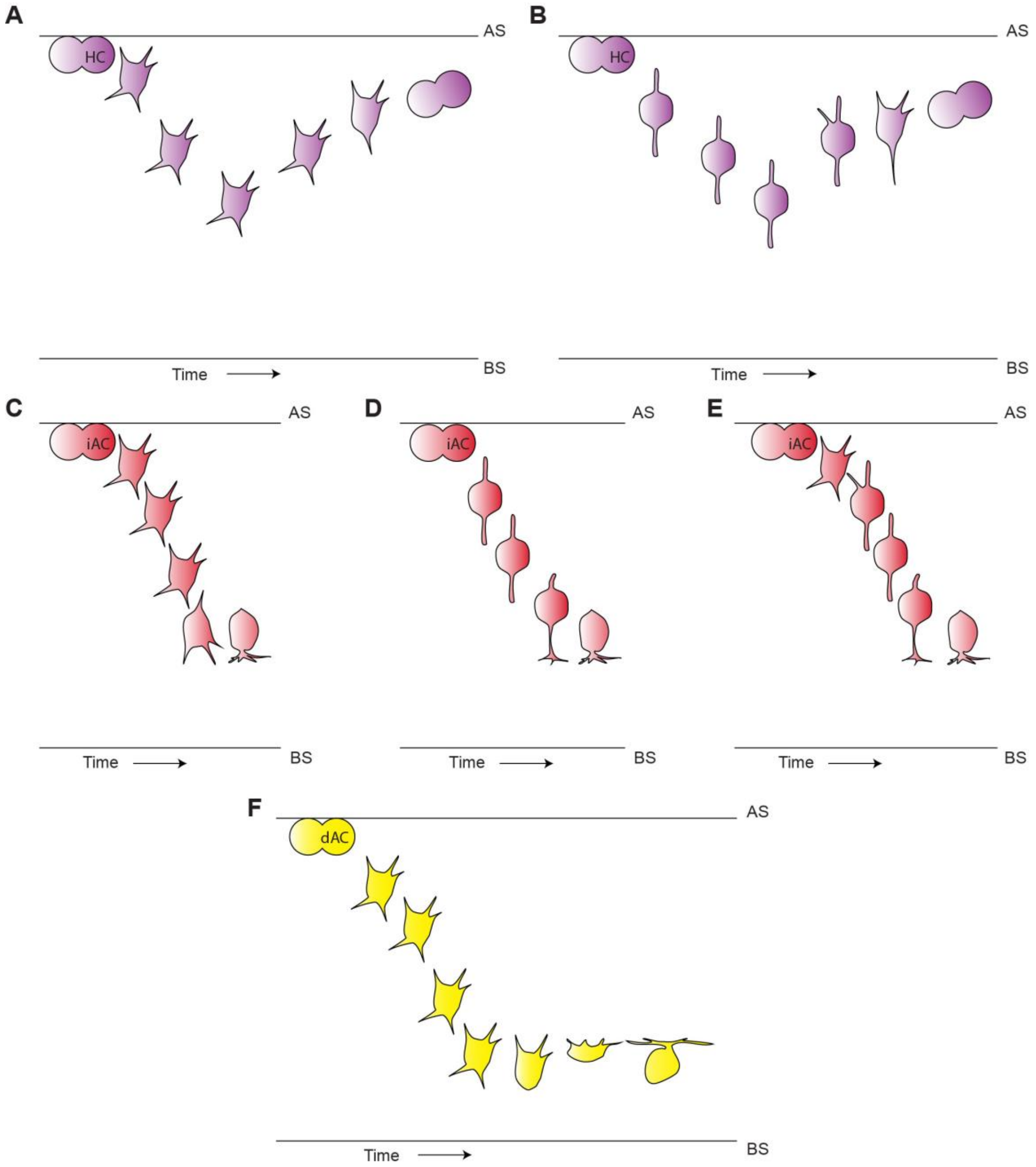

Fig S1: Models of RIN migration discussed in recent literature
A. After birth at the apical surface (AS), HCs detach from both the AS and basal surface (BS) of the retina, and undergo basally directed, multipolar migration. After migrating basally, HCs migrate apically via multipolar migration. B. HCs migrate basally via bipolar migration, but take on multipolar morphologies when migrating apically. C. iACs detach from both the AS and BS of the retina and undergo multipolar migration to their laminar destinations. D. iACs detach from both the AS and BS of the retina and undergo bipolar migration to their laminar destinations. E. iACs first detach from both the apical and basal surface of the retina and become multipolar, then, as they migrate basally to their final destinations, gradually prune their processes until they become unipolar. F. dACs detach from both the AS and BS of the retina and undergo multipolar migration to their laminar destinations, after which they polarize apically.

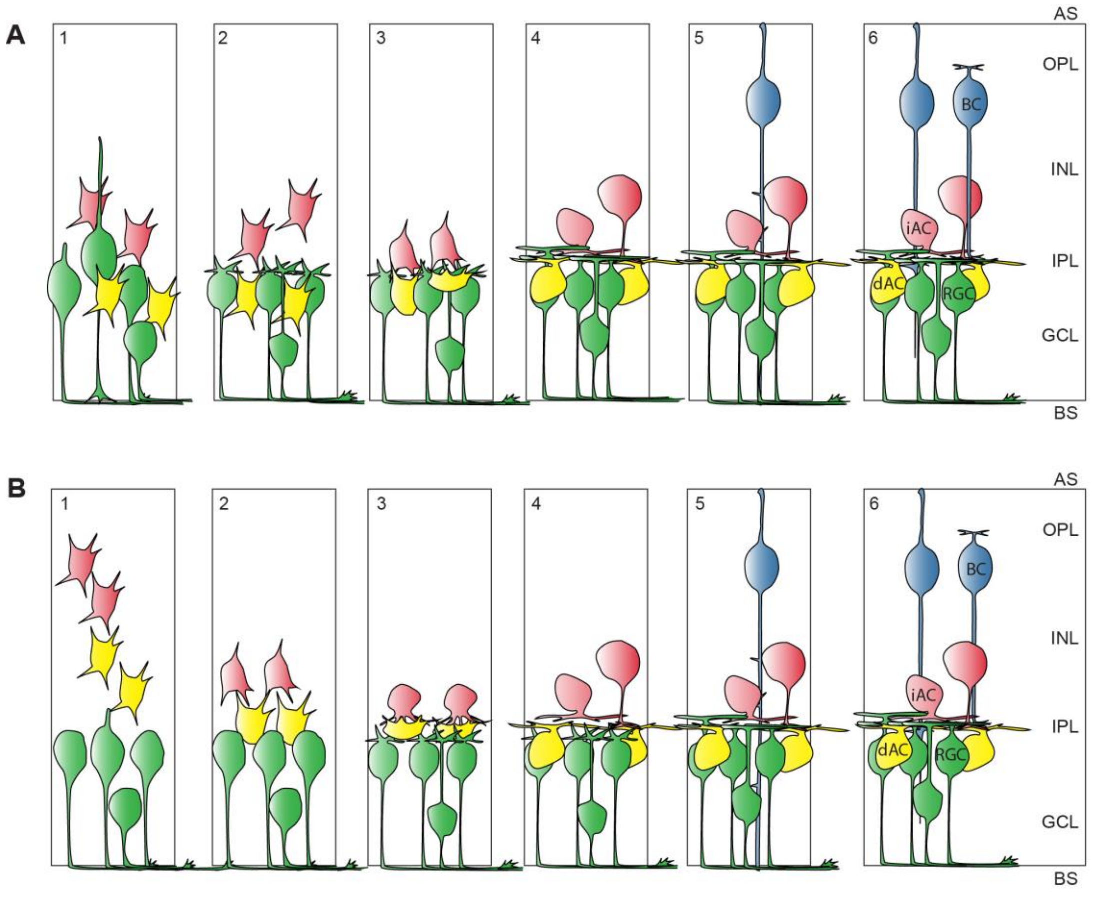

Fig S2: Models of IPL formation discussed in recent literature
A. 1. RGCs and ACs migrate in an overlapping sequence, resulting in interdigitated cell bodies. 2. The initiation of the IPL by RGC dendrites splits the iACs and the dACs. 3. ACs then direct their neurites towards RGC dendrites regardless of somal position. 4. RGCs and ACs stratify. 5-6. BCs direct their processes towards the IPL and stratify. B. 1. ACs arrive at a pre-formed RGC layer. 2. iACs and dACs initiate IPL formation by directing processes towards each other. 3. RGC dendrites accumulate beneath dACs 4. RGC dendrites bypass cell bodies of dACs and joins up with the iAC and dAC derived plexus. 5-6. BCs stratify after RGCs and ACs stratify.

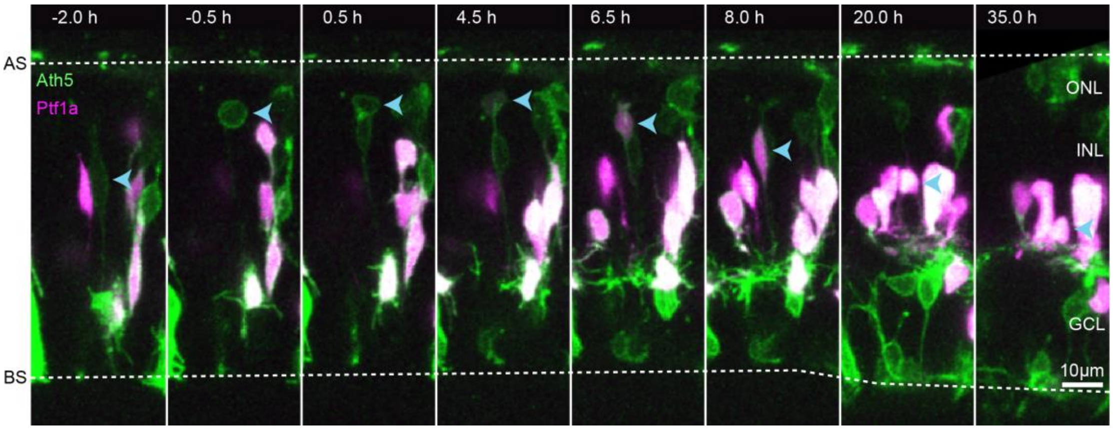

Fig S3: Occasionally, RINs are born near the region of the proto-OPL
Selected frames from a movie of an atypical RIN born near the region of the proto-OPL. Movie starts at ~48 hpf.

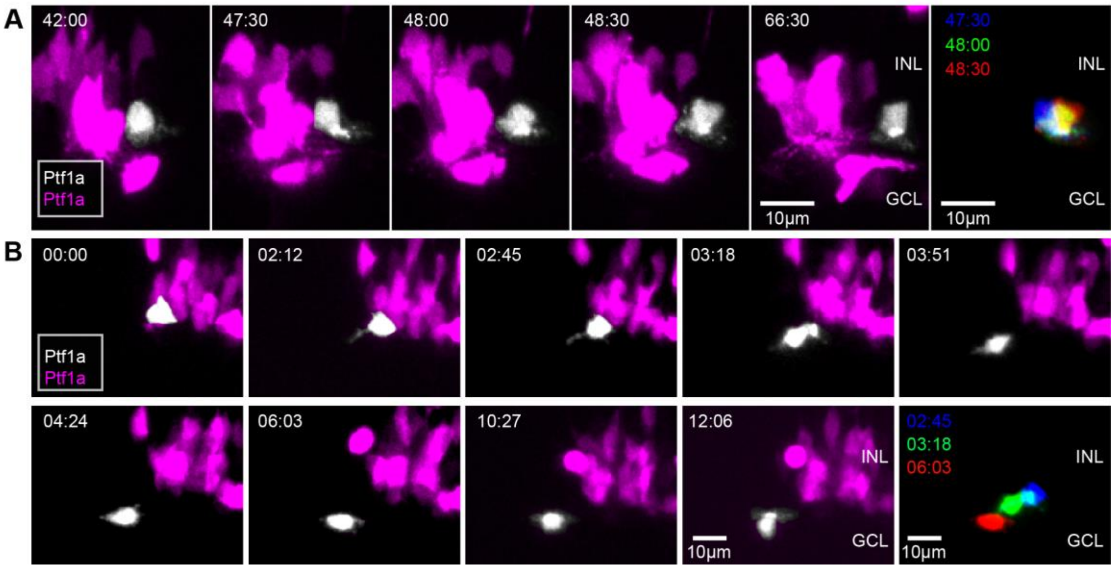

Fig S4. iACs and dACs undergo tangential migration near the proto-IPL
A. Example of iAC (white) migrating tangentially about half a somal length via a process extending laterally from the basal side of the cell. **B.** Example of a dAC (white) migrating tangentially via process extending from its flattened soma as it migrates basally into the GCL. Times are shown in h:min relative to the start of the movies. Movies start at ~48hpf.

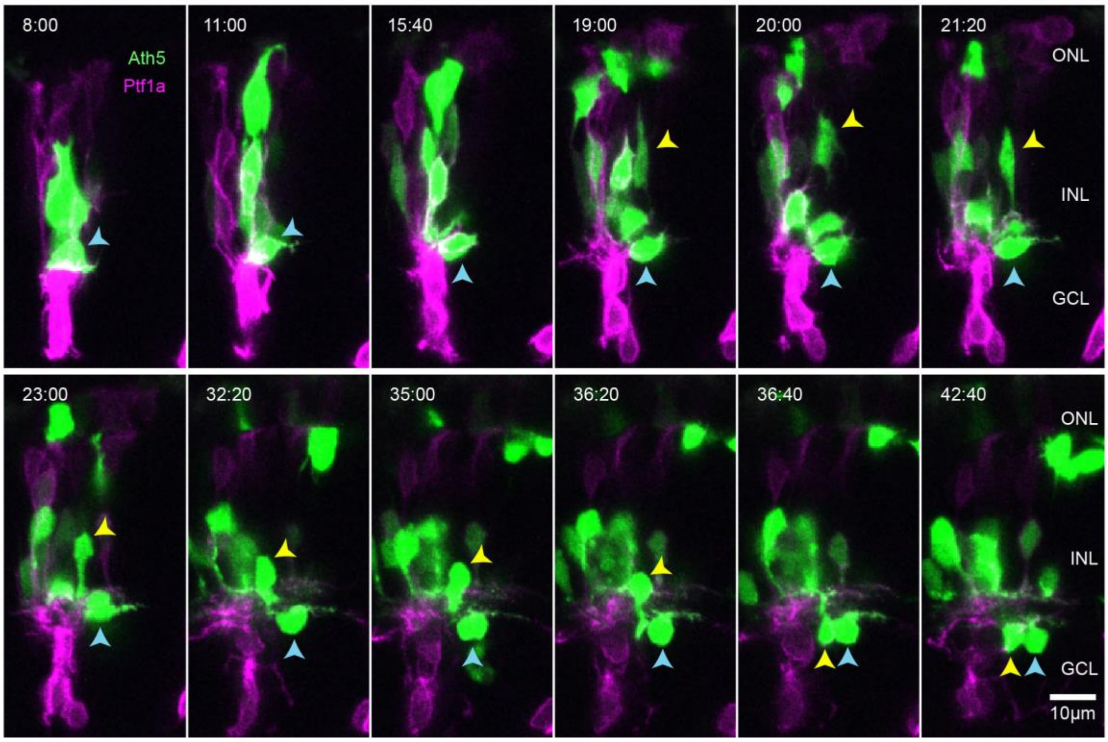

Fig S5: Example of an atypical dAC migrating into the GCL
Selected frames from a movie of a dAC (orange arrowheads) migrating into the GCL later than usual, when RGCs in the same clone are already clearly stratified. The dAC does not flatten, but squeezes through the forming IPL rapidly. For comparison, a dAC that migrated earlier is also indicated (blue arrowheads). Movie starts at ~48 hpf.

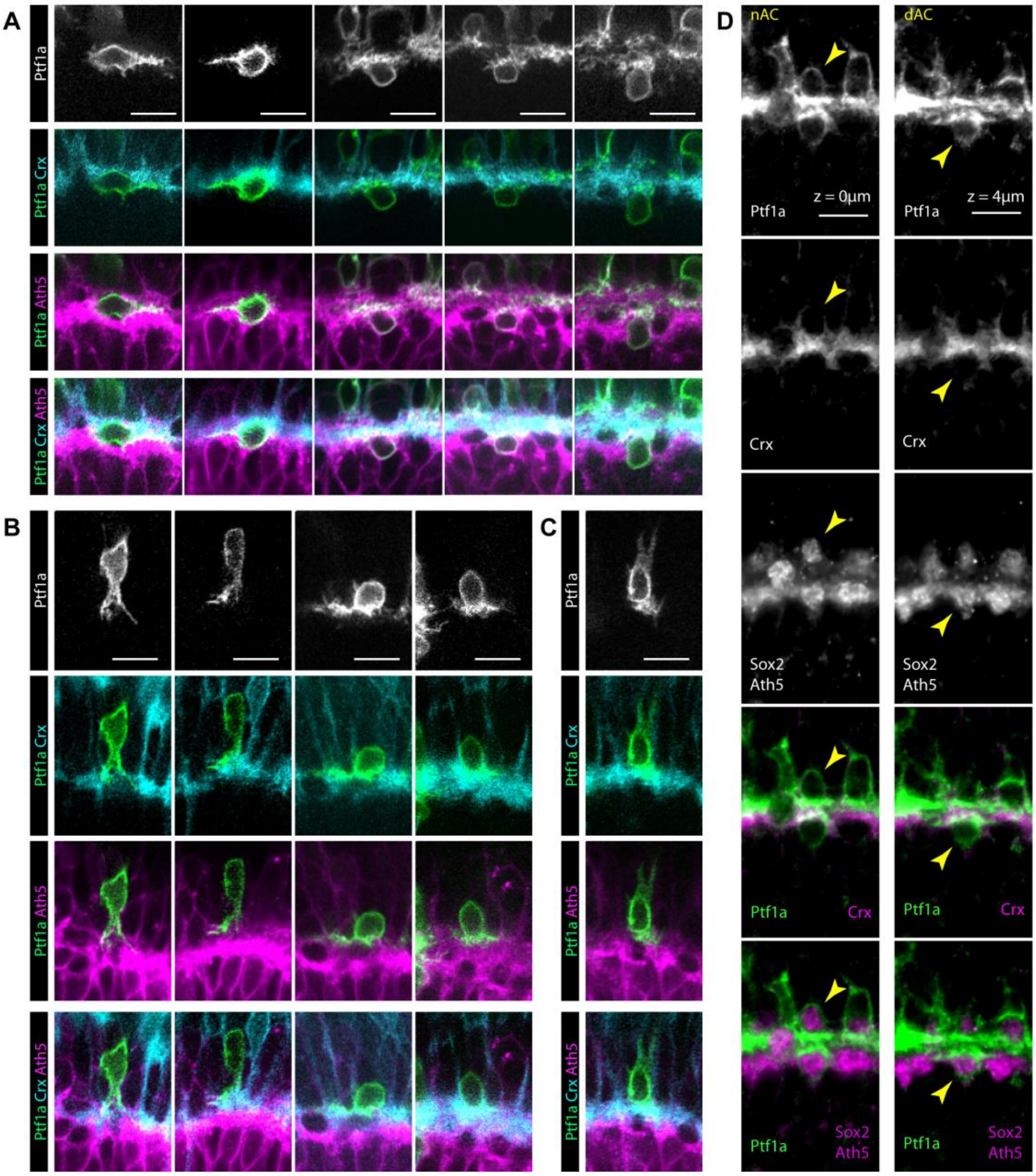

Fig S6: iACs and dACs differentially stratify in the proto-IPL
A. Isolated labelled dACs identified in 48-60 hpf SoFa2 retinas, where RINs are mosaically labelled with Ptf1a driven YFP, BCs are labelled using Crx driven CFP and RGCs are labelled using Ath5 driven RFP. Each column shows a different presumptive dAC. Images are arranged from left to right to suggest a possible sequence of migration. YFP labelled cells found basal to the BC plexus generally run lateral processes across the interface of the BC (cyan) plexus and the RGC plexus (magenta). **B.** Most isolated labelled iACs identified in the SoFa2 retina appear to initially stratify in the apical side of the BC plexus. Images are arranged from left to right to suggest a possible sequence of migration. **C.** Rarely, iACs appear to stratify in the middle of the BC plexus. Images are arranged from left to right to suggest a possible sequence of migration. **D.** The SoFa2 retina for starburst ACs using Sox2 antibody. A starburst iAC and a starburst dAC close to each other are seen to stratify on the apical side of the BC plexus, and at the interface between the BC plexus and RGC plexus, respectively.

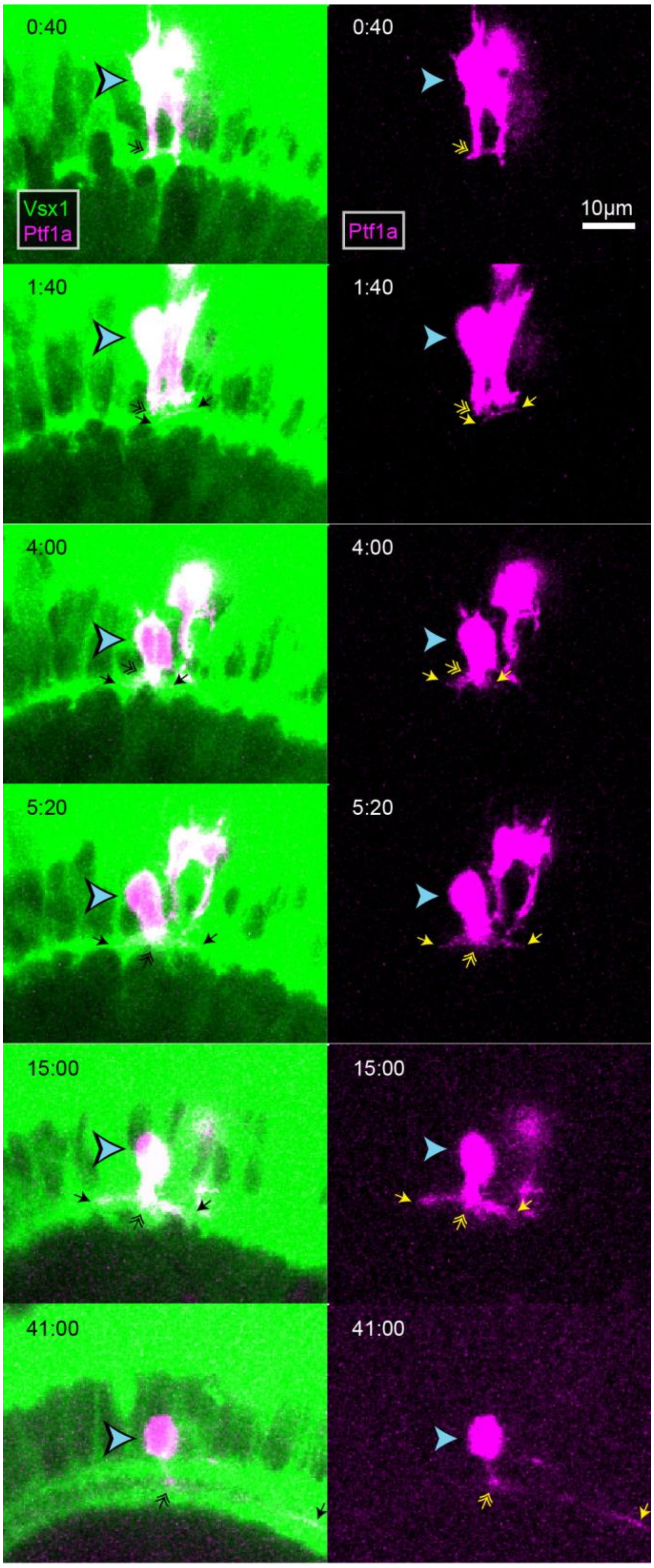

Fig S7: Example of a iAC stratifying in the middle of the BC plexus
Selected frames of a movie of a iAC (blue arrowheads) in the Vsx1GFP background. Feathered arrows indicate the iAC arbor's primary branch point. Solid arrows indicate the tips of the iAC's processes. Movie starts at ~55 hpf.

Supplementary Movies

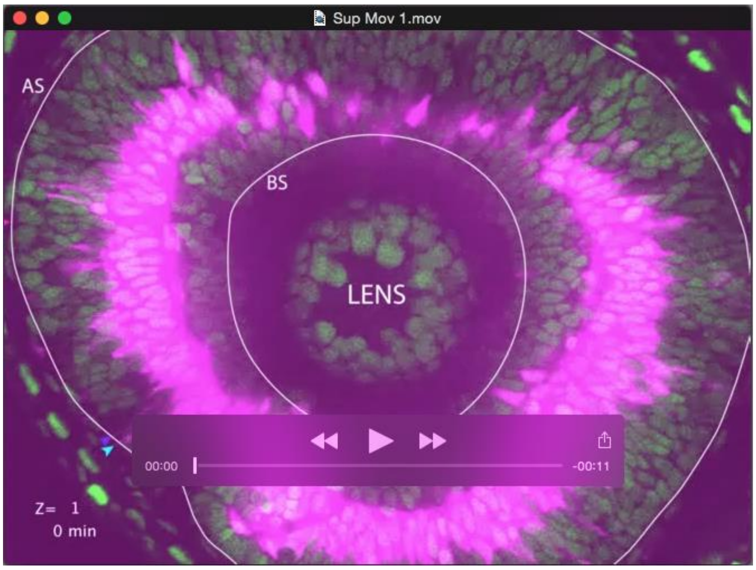

Supplementary Movie 1: RINs may switch from bipolar migration to multipolar migration

This movie shows the retina of a Ptf1a:DsRed embryo that was injected with GCaMP-NLS mRNA at the one cell stage. Imaging commenced ~45 hpf. The movie first runs through the z stack in the first time point, and it can be seen that RINs (magenta) in more apical regions of the retina appear to have elongated, smooth morphologies with apical processes (coloured arrows) attached to the apical surface of the retina (outside white line). GCaMP-NLS labels most cell nuclei (green), and it can be seen that RINs gather in a layer in the middle of the retina that is two to three cell somas thick. The movie then switches to extended focus and progresses forward in time, and it can be seen that, out of the RINs that turn on DsRed while they are in more apical regions of the retina, all of them retract their apical processes only when they have reached the middle of the retina. It is difficult to see the morphology of most RINs located in the middle of the retina due to density of labelling. However, those that can be distinguished from nearby RINs appear multipolar (coloured open circles). Multipolar RINs located in the apical region of the retina (coloured closed circles) are not apparent until RINs have spent some time gathering in the middle of the retina, and appear to be cells that are migrating apically. These cells are likely HCs, and some of them (eg. purple closed circle) can be seen to divide.

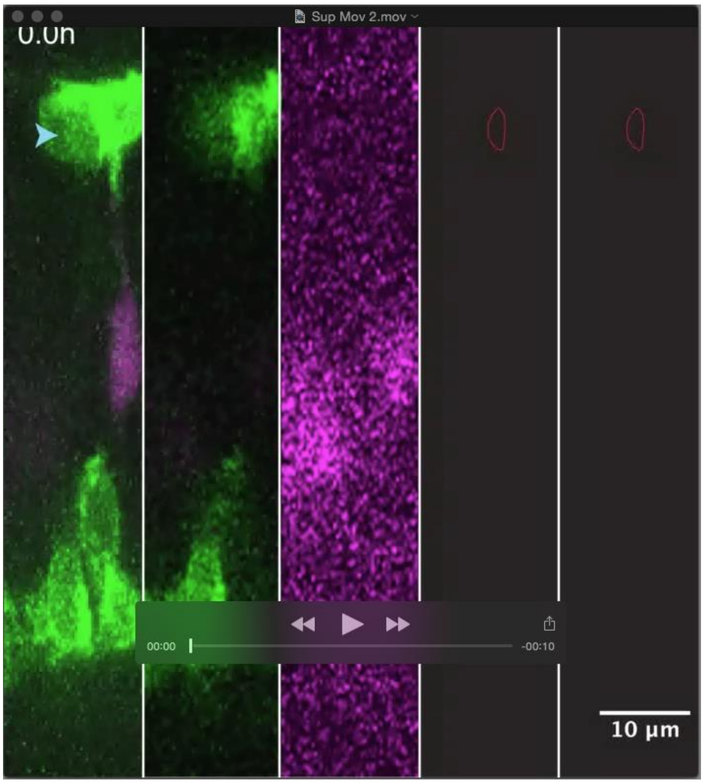

Supplementary Movie 2: RINs transition to bipolar morphologies immediately after birth

Cells are best visualized by going through the z-stack slice by slice. This movie shows how Fig 2D was made. The left-most panel shows the z-projection with both the magenta (Ptf1a) and green (Ath5) channels, the second left panel shows the single z slice with both channels, the third panel shows the single z slice with the magenta (Ptf1a) channel only (overexposed in order to see the position of the apical process), the forth panel shows the Illustrator trace of the cell in that slice, and the fifth and right-most shows the cumulative traces. Time is shown as hours from cell birth. Z-slices are spaced 1µm apart.

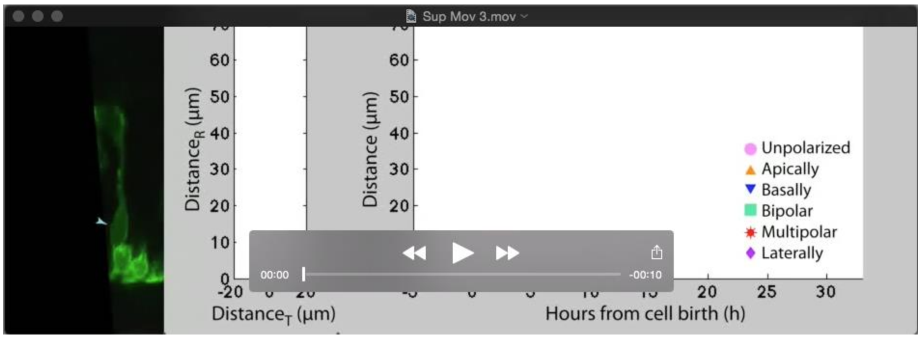

Supplementary Movie 3: Example of HC migration
Ath5:gapRFP::Ptf1a:GFP blastomeres were transplanted into wild type embryos at 4 hpf to create mosaic embryos where only a few cells are fluorescently labelled, allowing the morphology of individual RINs to be observed. In the left panel, the HC (blue arrow) shown initially expresses Ath5 (green) only, and later turns on Ptf1a (magenta). Time is shown relative to cell birth. The middle panel shows the movement of the HC over time, with the cell's radial position relative to the basal surface of the retina shown on the y-axis and the tangential position relative to position at birth shown on the x-axis. On the right panel is the graph showing the distance of the HC relative to basal surface of the retina over time. The black line represents the middle of the HC's soma, the greyed area represents the apical and basal extent of the HC's soma. The markers on the black line show how the cell's morphology was classified at that particular time point. Movie starts at ~44 hpf.

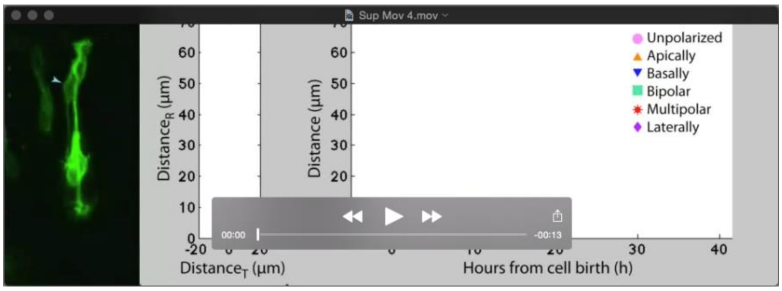

Supplementary Movie 4: Example of iAC migration
iAC corresponding to Fig 2B. Panels are laid out the same way as Sup Movie 2. Movie starts at ~44 hpf.

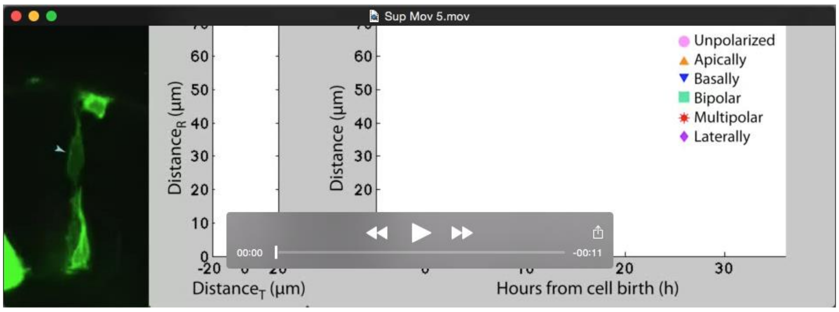

Supplementary Movie 5: Example of dAC migratiom
dAC corresponding to Fig 2C. Panels are laid out the same way as Sup Movie 2. Movie starts at ~44 hpf.

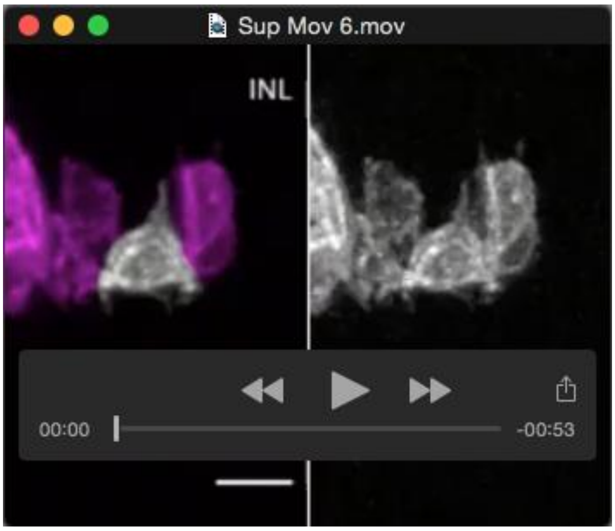

Supplementary Movie 6: dACs undergo a specific sequence of morphological changes to become apically polarized
Movie of the dAC shown in Figure 4B captured in a Ptf1a:Gal4::UAS:YFP embryo. Time is written as h:min. In the left panel, the cell of interest has been pseudocoloured white using Photoshop. In the right panel, the uncoloured image is shown. The movie pauses at various timepoints and goes through the z-stack slice by slice. Z-slices are spaced 0.75µm apart. Movie starts at ~48 hpf.

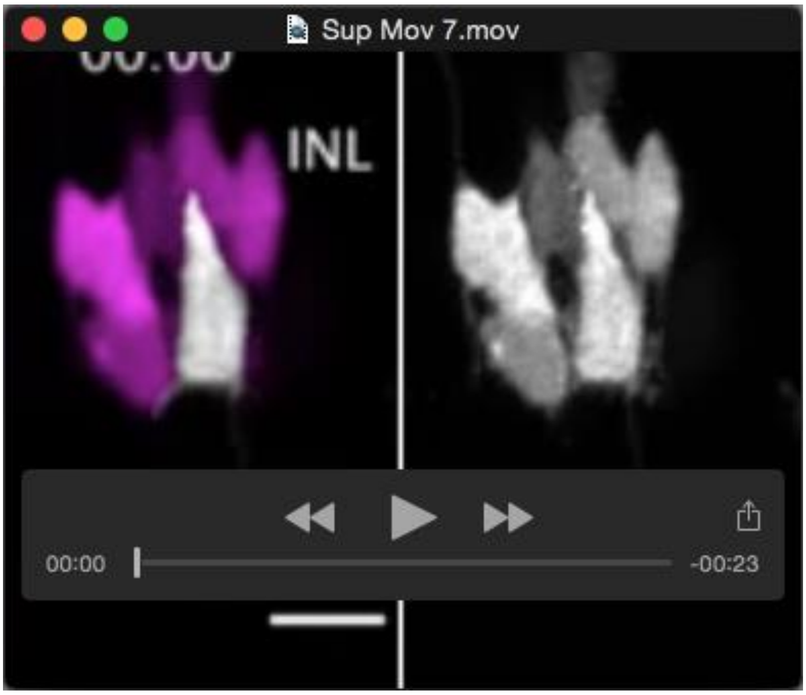

Supplementary Movie 7: dACs undergo a specific sequence of morphological changes to become apically polarized
Movie of the dAC shown in Fig 4C. Cells from Ptf1a:DsRed embryos were transplanted into a WT background. In the left panel, the cell of interest has been pseudocoloured white using Photoshop. In the right panel, the uncoloured image is shown. Time is written as h:min. Movie starts at ~48 hpf.

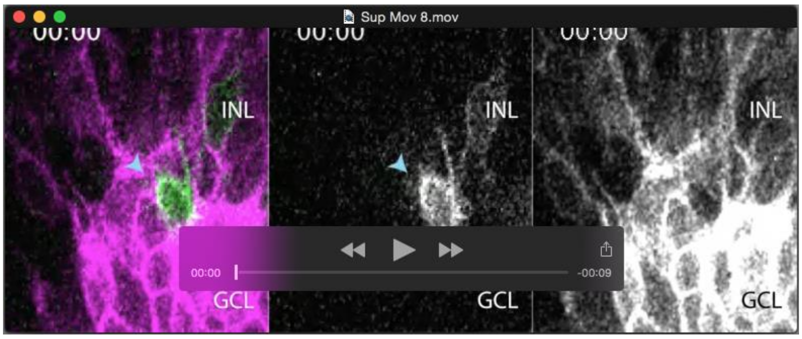

Supplementary Movie 8: During early stages of IPL formation, iACs stratify at a plane apical to the RGC plexus
Movie of the iAC shown in Fig 9A. Time is written as h:min. The left panel shows both the Ptf1a:YFP signal (green) and the Ath5:gapRFP signal (magenta). The iAC is labelled using blue arrows. The middle panel shows the Ptf1a:YFP signal (green) only. The iAC is labelled using blue arrows. The right panel shows the Ath5:gapRFP signal (magenta) only. Movie starts at ~50hpf.

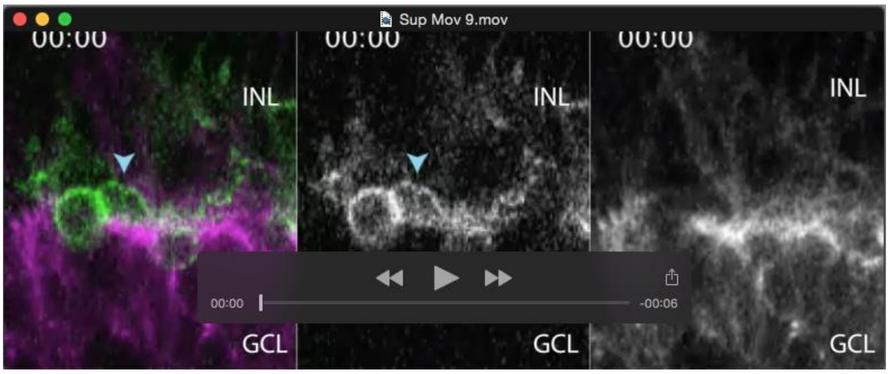

Supplementary Movie 9: dACs stratify at the apical side of the RGC plexus and migrate through the RGC plexus.
Movie of dAC shown in Fig 9B. Time is written as h:min. The left panel shows both the Ptf1a:YFP signal (green) and the Ath5:gapRFP signal (magenta). The dAC is labelled using blue arrows. The middle panel shows the Ptf1a:YFP signal (green) only. The dAC is labelled using blue arrows. The right panel shows the Ath5:gapRFP signal (magenta) only. Movie starts at ~50hpf.

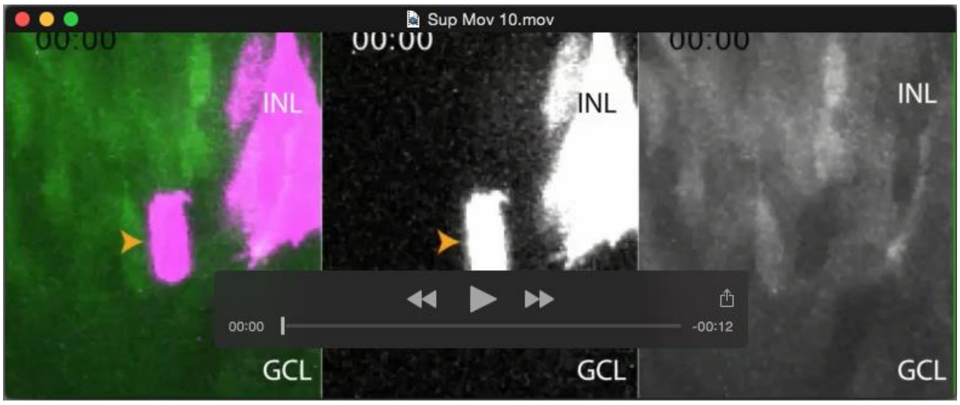

Supplementary Movie 10: During early stages of IPL formation, most iACs stabilize processes at the apical side of the BC plexus
Movie of the iAC shown in Fig 9C. Movie starts at ~50 hpf. Time is written as h:min. The left panel shows both the Ptf1a:DsRed signal (magenta) and the Vsx1:GFP signal (green). The iAC is labelled using blue arrows. The middle panel shows the Ptf1a:DsRed signal (magenta) only. The iAC is labelled using blue arrows. The right panel shows the Vsx1:GFP signal (green) only.

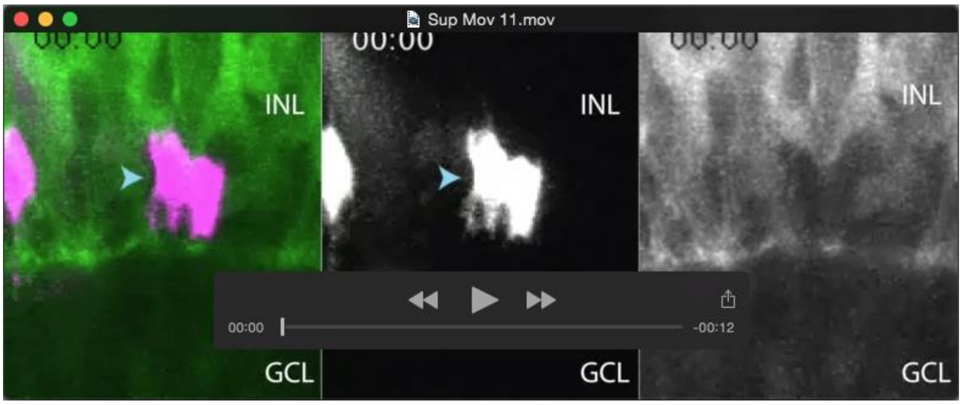

Supplementary Movie 11: DACs migrate through the BC plexus and stratify at the basal side of the BC plexus

Movie of dAC shown in Fig 9D. Time is written as h:min. The left panel shows both the Ptf1a:DsRed signal (magenta) and the Vsx1:GFP signal (green). The dAC is labelled using blue arrows. The middle panel shows the Ptf1a:DsRed signal (magenta) only. The dAC is labelled using blue arrows. The right panel shows the Vsx1:GFP signal (green) only. Movie starts at ~50 hpf.

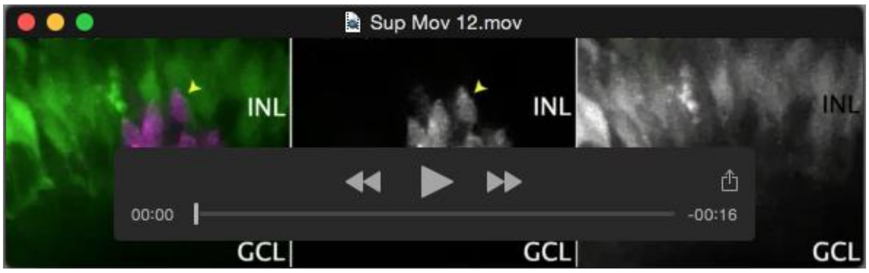

Supplementary Movie 12: iACs and dACs migrate tangentially along the interfaces of the BC plexus

Movie of a iAC (blue arrow) and a dAC (yellow arrow) migrating tangentially away from each other at either side of the BC plexus. The movie starts around 60hpf. Time is written as h:min. Movie starts at ~50 hpf.
